# Supplementary figures and images for: From trivalent to quadrivalent influenza vaccines: Public health and economic burden for different immunization strategies in Spain
Source: PLoS One. 2020 May 21;15(5):e0233526. doi: 10.1371/journal.pone.0233526 (PMC7241783; doi:10.1371/journal.pone.0233526)

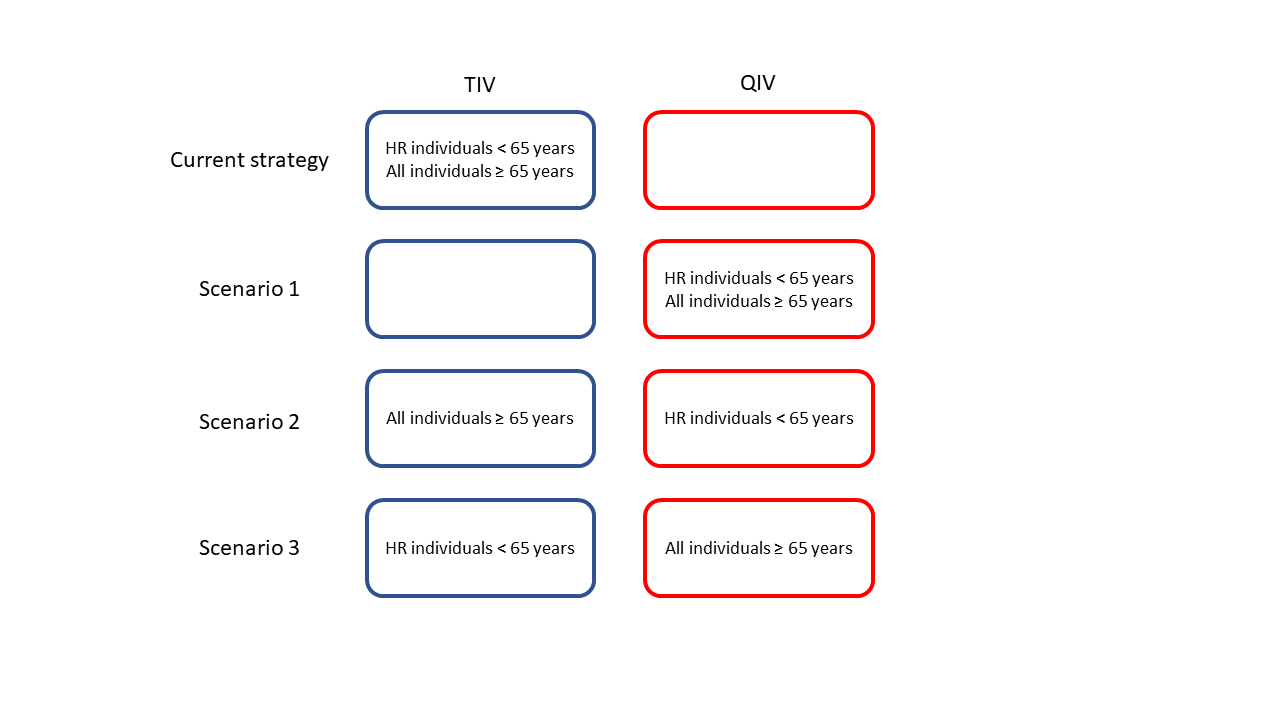

Supplement: S1 Fig — (TIF) [file pone.0233526.s001.tif]
